# Combined Transcriptome and Proteome Analysis to Elucidate Salt Tolerance Strategies of the Halophyte *Panicum antidotale* Retz

**DOI:** 10.3389/fpls.2021.760589

**Published:** 2021-11-02

**Authors:** Tabassum Hussain, Hina Asrar, Wensheng Zhang, Bilquees Gul, Xiaojing Liu

**Affiliations:** ^1^Chinese Academy of Sciences Engineering Laboratory for Efficient Utilization of Saline Resources, Center for Agricultural Resources Research, Institute of Genetics and Developmental Biology, Chinese Academy of Sciences, Shijiazhuang, China; ^2^Dr. M. Ajmal Khan Institute of Sustainable Halophyte Utilization, University of Karachi, Karachi, Pakistan

**Keywords:** halophyte, molecular pathway, salt tolerance, integrated transcriptome and proteome, monocot

## Abstract

*Panicum antidotale*, a C4 monocot, has the potential to reclaim saline and drylands and to be utilized as fodder and forage. Its adaptability to survive saline stress has been proven with eco-physiological and biochemical studies. However, little is known about its molecular mechanisms of salt tolerance. In this study, an integrated transcriptome and proteome analysis approach, based on RNA sequencing and liquid chromatography tandem mass spectrometry (LC-MS/MS), was used to identify the said mechanisms. Plants were treated with control (0 mM), low (100 mM), and high (300 mM) sodium chloride (NaCl) treatments to distinguish beneficial and toxic pathways influencing plant biomass. The results indicated differential expression of 3,179 (1,126 upregulated/2,053 downregulated) and 2,172 (898 upregulated/1,274 downregulated) genes (DEGs), and 514 (269 upregulated/245 downregulated) and 836 (494 upregulated/392 downregulated) proteins (DEPs) at 100 and 300 mM NaCl, respectively. Among these, most upregulated genes and proteins were involved in salt resistance strategies such as proline biosynthesis, the antioxidant defense system, ion homeostasis, and sugar accumulation at low salinity levels. On the other hand, the expression of several genes and proteins involved in the respiratory process were downregulated, indicating the inability of plants to meet their energy demands at high salinity levels. Moreover, the impairments in photosynthesis were also evident with the reduced expression of genes regulating the structure of photosystems and increased expression of abscisic acid (ABA) mediated pathways which limits stomatal gas exchange. Similarly, the disturbance in fatty acid metabolism and activation of essential ion transport blockers damaged the integrity of the cell membrane, which was also evident with enhanced malondialdehyde (MDA). Overall, the analysis of pathways revealed that the plant optimal performance at low salinity was related to enhanced metabolism, antioxidative defense, cell growth, and signaling pathways, whereas high salinity inhibited biomass accumulation by altered expression of numerous genes involved in carbon metabolism, signaling, transcription, and translation. The data provided the first global analysis of the mechanisms imparting salt stress tolerance of any halophyte at transcriptome and proteome levels.

## Introduction

Modern agriculture practices have aggravated soil salinity, making it to become a globally complex environmental problem. To date, approximately 20% of the arable land around the world is affected by salts and a further increase to as much as 50% of cultivated land being salt damaged is expected by the year 2050 (Thenkabail, 2010). Salty soils impede the growth and yield of several cash crops by combining osmotic stress and ion toxicity. While the exposure of the former disturbs plant water relationships, the latter affects various physiological functions. Moreover, salinity-induced secondary stresses, such as excessive reactive oxygen species (ROS), limit cellular metabolism by damaging biomolecules (Zhu, 2016). High concentrations of salt in the soil can even lead to plant death. Thus, improving the salt tolerance of crops has become an important research topic in alliance with the sustainable development goal of providing food to our rapidly growing population (Assembly, 2015).

Numerous reports have offered insights into the physiological and biochemical adaptations of plants to cope with salinity (Duarte et al., 2015; Zhou et al., 2019). These adaptations are a consequence of rearrangement at the molecular level—starting from stress-specific transcription regulation to mRNA processing, protein synthesis, post-translational modifications, and metabolite levels (Reynolds et al., 2005; Zhang and Shi, 2013). Halophytes, the natural salt-tolerant plants, serve as a repository of salt-responsive genes and therefore, are ideal candidates to study salt tolerance strategies (Rozema and Schat, 2013; Yuan et al., 2019). Designing such studies will support the strategic decision to escalate crop yields on salt-laden lands by stakeholders including farms, environmental groups, government agencies, health organizations, and researchers.

The establishment of next-generation sequencing (NGS) technologies coupled with other omics tools facilitated the identification of key genes expressed in stressed environments. For instance, the modulation of genes involved in the controlled uptake of salts, the compartmentalization of toxic ions, the synthesis of compatible solutes, and the scavenging of ROS was characterized and several transgenic lines for economically important crops were developed (Zhang et al., 2011; Huang et al., 2012; Lv et al., 2018). Though plant biologists have deciphered the stress-related molecular mechanisms, the limited salt resistance achieved for genetically modified crops prevents us from claiming a complete understanding of plant response to saline environments (Arzani, 2008). It instead reflects the multigenic nature of the salt tolerance trait and the intricate interactions that exist among various genes. Regarding the functional significance of expressed genes in salt tolerance, not only alterations in the relative abundance of mRNAs, but also changes in proteome profile, protein-protein interactions, and post-translational modifications deserve to be studied (Soda et al., 2015; Jha et al., 2019).

Recent advances in omics approaches, i.e., genomics, transcriptomics, proteomics, and metabolomics, have allowed researchers to quantify salt resistance strategies at every level of gene expression. Contrarily, the available literature reports the use of a single “omic” tool, such as transcriptome which is the most routinely used tool, to measure the differentially expressed genes. This practice is alarming as a weak correlation exists between different steps of gene expression, e.g., the abundance of transcript and protein (Maier et al., 2009; Vogel and Marcotte, 2012; Lai et al., 2020). However, this frequent inconsistency suggests the need for complementary analysis for further validation of salt-responsive genes and metabolic pathways. In addition to endorsing transcript-proteome relationships, such analyses can identify changes of correlation between control and stress-treated plants, helping us to better comprehend the molecular mechanisms underpinning salt tolerance. For instance, researchers working on yeast indicated correlations among mRNA and protein to be strong at severe stress while weak at low stress (Halbeisen and Gerber, 2012). Therefore, the tremendous increase in the number of multi-omics-based studies in the last couple of years is no surprise. However, following the conventional pattern of information collection, these studies are set for model plants and salt-sensitive crops (Peng et al., 2018; Arefian et al., 2019; Meng et al., 2020). Analysis of the gene expression of halophytes with a coordinated omics approach, on the other hand, has more potential to dissect the master regulators of salt stress physiology and biochemistry. The scarcity of such data for halophytes also raises concerns: are different stages of gene expression unrelated or just understudied in these plants? Answers to these questions are crucial to develop salt resistance in sensitive plants.

*Panicum antidotale*, a C4 perennial of the family Poaceae, is a widely distributed halophyte along with the dry and saline lands of Asia. Because of its utilization as fodder/forage in different regions of the world and its phylogenetic relationship with food crops, such as millet, rice, wheat, and barley, this grass has attracted researchers to dissect salt resistance pathways. Our previous studies (Hussain et al., 2015, 2020) have accumulated a great deal of information on physiological and biochemical adaptations to perceive the survival potential of this plant in saline environments. For instance, a better performance at low salinity while survival up to seawater salinity with a marginal mortality rate. The execution of salt tolerance strategies, such as maintained plant water status, use of sodium ion (Na^+^) as a cheap osmoticum, accumulation of compatible solutes including storage carbohydrates and proline, and protection against oxidative damage, however, required a surplus amount of energy. Since photosynthetic performance and antioxidative defense mechanisms were disturbed by the high concentration of salts, the reallocation of available energy resources from growth to salt tolerance mechanisms sustained its prolonged survival in those conditions. Despite this plethora of information, the molecular mechanisms underlying salt tolerance of *P. antidotale* remain largely uncharacterized. Considering this, the current study was designed to use a coordinated approach, i.e., a conjoint analysis of transcriptome and proteome profiles, to identify master regulators of salt stress. The purpose of this study was to relate eco-physiological responses to candidate genes playing critical roles in stress response. Since the productivity of halophytes differs with levels of salt in the growth medium, the zero, low, and high salinity treatments were compared with identify molecular pathways related to either improved or inhibited growth. To the best of our knowledge, this is the first study in which the interacting molecular partners of salt stress response of any halophyte will be identified. Besides, the data generated hereby will serve as a blueprint for the transcriptome and proteome dynamics of a salt-resistant monocot. The applied significance of this study will be embraced by the transfer of salt tolerance traits to non-halophyte crops.

## Materials and Methods

### Plant Materials and Salinity Treatments

The seeds of *P. antidotale* were germinated at 25°C with a photoperiod of 14 h. Three leaf stage seedlings were grown in pots (15 cm × 22 cm) containing Quartz sand in a glasshouse for 21 days under controlled conditions (25°C/14°C, 14/10-h day/night regimes, 600 ± 50 μmol photon m^–2^ s^–1^, 45–65% humidity). The plants were supplied with half-strength Hoagland’s nutrient solution (Epstein, 1972) which was replaced every 3 days to avoid nutrient deficiencies. After 4 weeks, plants were exposed to 0 (control), 100 (low) and 300 mM (high) sodium chloride (NaCl). The treatments were induced gradually by the addition of 50 mM NaCl twice a day unless desired concentrations were achieved. Three replicates were used for each treatment. Plants were harvested after 4 weeks of treatments and fresh weight was measured immediately. However, malondialdehyde (MDA) and chlorophyll contents were measured as described previously (Hussain et al., 2020). Leaf samples were immediately immersed in liquid nitrogen before being stored at −80°C for transcriptome and proteome analysis.

### RNA Extraction, cDNA Library Construction, and Sequencing

Total RNA of the leaf samples was extracted with the RNAprep Pure Plant Kit (Tiangen Biotech Co., Ltd., Beijing, China) following the instructions of the manufacturer and treated with RNase-free DNase I to remove any contaminant of DNA. The quality and quantity of extracted RNA were checked with agarose (1.2%) gel electrophoresis and verified at 260 and 280 nm using a NanoDrop ND-1000 spectrophotometer (Thermo Fisher Scientific, Inc., Waltham, MA, United States). Poly (A) mRNA was enriched from total RNA by using NEBNext^®^ Poly(A) mRNA Magnetic Isolation Module (New England Biolabs, Ipswich, MA, United States) and fragmented into short pieces by chemical. The obtained fragments were taken as a template for the first- and second-strand cDNA synthesis. The resulting cDNA fragments, after their purification with Qiaquick PCR purification kit (Qiagen, Hilden, Germany), were ligated to the sequencing adapters. The fragments of appropriate size were selected by using agarose gel electrophoresis to be used as a template for PCR amplification. Finally, nine cDNA libraries were constructed and sequenced on a flow cell using Illumina HiSeq. 2500 High-throughput Sequencing machine, United States.

### *De novo* Transcriptome Assembly and Data Analyses

The raw data were filtered by trimming adapters and removing low-quality sequences to obtain a clean read. Then, Q20, Q30, and GC-content of cleaned data were calculated. For each library, first clean short reads were assembled into contigs with longer contiguous sequences based on their overlap regions. Contigs from other transcripts were pooled, clustered, and assembled by using the Trinity software^1^ (Grabherr et al., 2011). The obtained sequences that can no longer be extended on either side were referred to as “unigenes”. All assembled unigenes were annotated with GetORF from the EMBOSS package (Rice et al., 2000). The predicted Open Reading Frame (ORF) was used for BLAST searches. For functional annotation, unigene sequences were aligned to publicly available databases including the National Centre for Biotechnology Information (NCBI) non-redundant protein (Nr), SwissProt, Gene Ontology (GO)^2^, EuKaryotic Orthologous Groups (KOG), and the Kyoto Encyclopedia of Genes and Genomes (KEGG) pathway^3^ using BLASTX algorithm with 10^–5^ as *E*-value cut-off point.

### Simple Sequence Repeats Detection

Simple sequence repeats were identified using MISA (microsatellite identification tool) (Thiel et al., 2003) and filtered to represent unique polymorphisms. The minimum number of nucleotide repeats was 10 for mononucleotide repeats, seven for dinucleotide repeats, and five for other repeats, i.e., tri-, tetra-, penta-, and hexanucleotide repeats. The maximum number of bases interrupting a compound SSR was set to 100 base pairs. Primer design was performed in batch with Primer3 (Untergasser et al., 2012) using default parameters and Perl scripts.

### Analysis of Differentially Expressed Genes

The expression level of each transcript was estimated with the fragments per kilobase of gene per million mapped reads (FPKM) method, which eliminates the effect of differences in gene length and sequencing discrepancies. FPKM values were calculated with SEMRSEM software. False-positive and false-negative errors were corrected by calculating the false discovery rate (FDR) values (Benjamini and Yekutieli, 2001). Differential expression analysis of genes was determined using EBSeq sofware27. The False Discovery Rate (FDR) and log_2_FC (log of fold change) were calculated for all genes. DEGs were selected by using an FDR value ≤ 0.05 and a log_2_ fold-change > 1 as a threshold value. The log2-transformed FPKM values of DEGs were used to generate a heat map by Heml (Deng et al., 2014). The GO terms and the KEGG pathways, enriched within the DEGs, were identified by agriGO (Du et al., 2010) and FatiGO (Al-Shahrour et al., 2004) software, respectively.

### Protein Extraction, Digestion, and Labeling

Plant samples were ground to powder with liquid nitrogen and proteins were extracted using a lysis buffer (7 M urea, 2 M thiourea, 0.1% CHAPS, 0.1% protease inhibitor cocktail). The supernatant was collected after centrifugation at 14,000 × *g* for 30 min at 4°C, and total soluble protein concentration was estimated by Bradford assay using the Quick Start^TM^ Bradford reagent (Bio-Rad, Hercules, CA, United States) and bovine serum albumin as a standard. The quality and concentration of protein were verified by running sodium dodecyl sulfate-polyacrylamide gel electrophoresis (SDS-PAGE) (12% gels). For each protein sample, 100 μg of proteins were reduced, alkylated, trypsin digested, and labeled using the standard TMT^®^ kit (Thermo Fisher Scientific) according to the instructions of the manufacturer. N (nitrogen) and C (carbon) samples were labeled with the tags 127–131. Three independent biological replicates with three technical replicates were performed.

### Liquid Chromatography Coupled With Tandem Mass Spectrometry (LC-MS/MS) and Protein Quantification

The fractionated peptides were analyzed using a Thermo Q-Exactive mass spectrometer (Thermo Fisher Scientific). Raw data were processed with Proteome Discover version 1.4 (Thermo Fisher Scientific) and searched with Mascot software 2.3.02 (Matrix Science, London, United Kingdom) against a personalized transcriptome database for *P. antidotale*, with a precursor mass tolerance of 15 ppm, a fragment ion mass tolerance of 20 mm, and strict trypsin specificity. This allowed up to two missed cleavages, carboxyamidomethylation modification on cysteine residues as fixed modification, and oxidation of methionine residues and phosphorylation of serine, threonine, and tyrosine residues as variable modifications. Proteins with at least one unique peptide and a threshold of FDR having a *p*-value of < 0.01 were considered positive identification. A fold-change of ≥ 1.2 (*p* < 0.05) was taken as a threshold to identify differentially expressed proteins (DEPs).

### Functional Classification, Enrichment, and Pathway Analysis

Proteins were examined for functional annotation by using the Blast2GO program against the non-redundant (NR) protein database. Differentially expressed proteins (DEPs) were subjected to GO and KEGG significant enrichment analysis by using the hypergeometric test (*p* ≤ 0.05). The heat map was exhibited using the “heatmap” R-package. The protein-protein interaction (PPI) networks were constructed using the String program.

### Statistical Analysis

All the values reported in this study are an average of three replicates. We used SPSS software version 19, United States for the one-way ANOVA of the data. The difference was considered statistically significant at *P* < 0.05. Significance differences in transcripts expression were examined with Cuffdiff (Trapnell et al., 2013) (*P* < 0.05). Log2-transformed fold changes from three replicates were used for the analysis.

## Results

### Response to Salinity Treatments

**FIGURE 1:**
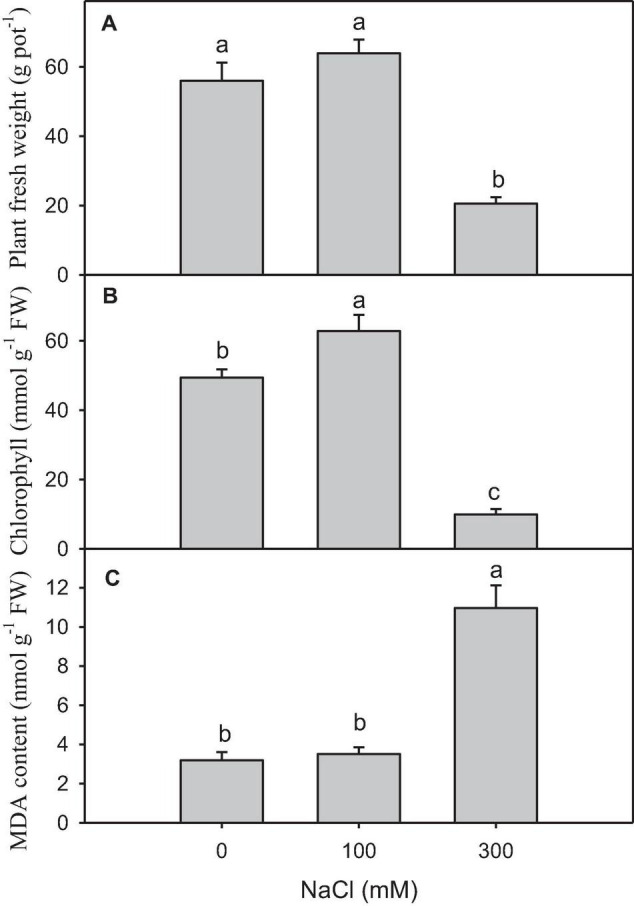
Physiological and biochemical changes in salt-treated *Panicum antidotale*. **(A)** The fresh weight of plants **(B)** The chlorophyll content in the leaves and **(C)** malondialdehyde (MDA) content in plants treated with 0, 100, and 300 mM NaCl (*n* = 3 ± *SD*). The different letters present the significant difference among treatments at the *P* < 0.5 after the Bonferroni *post hoc* test.

High salinity caused a significant reduction in plant growth and chlorophyll content compared with other treatments (Figures 1A,B). The content of MDA, which was the final product of lipid peroxidation, significantly increased in the plants treated with high salinity (Figure 1C).

### Sequencing and *de novo* Transcriptome Assembly

**FIGURE 2:**
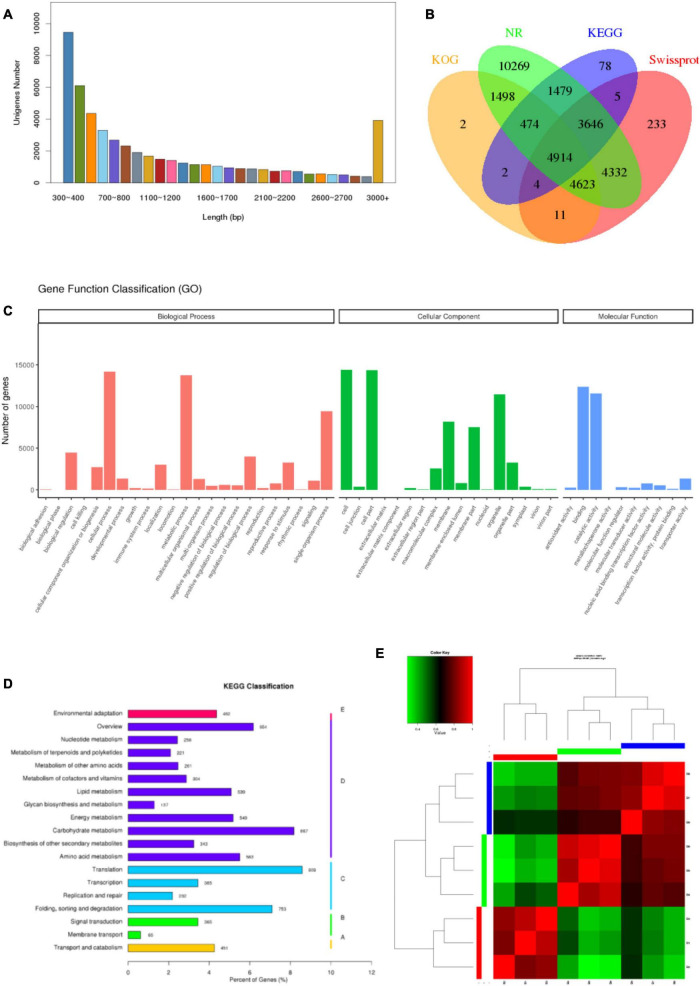
Characteristics of unigenes and samples. **(A)** Pattern of length distribution in unigenes of *P. antidotale.* **(B)** The proportion of transcripts annotated to different databases at the same time. **(C)** Distribution of identified genes as per Gene Ontology (GO) classification. **(D)** Distribution of identified genes as per Kyoto Encyclopedia of Genes and Genomes (KEGG) classification. **(E)** Correlation analysis between the samples.

To get a global overview of *P. antidotale* transcriptome response under salinity, RNA sequencing of three biological replicates from the control (B1, B2, and B3), low salinity (B4, B5, and B6), and high salinity (B7, B8, and B9) treated samples was performed. About 204.70 million reads were yielded in total with 125 base pairs for both paired ends. The clean reads (61.41 Gb data) were obtained after removing (i) adaptor sequences, (ii) low-quality reads (*Q*-value < 20), and (iii) reads containing more than 10% ambiguous N bases. Table 1 represents the number and size of clean reads in each sample. The GC content was approximately 55% in the nine samples and more than 94% Q30 bases were acquired. Comparable data were observed for the biological replicates. The filtered clean reads were assembled using the Trinity program. Subsequent to isoform detection, a total of 51, 835 unigenes (N50 value = 1,905) were generated. The mean size of these unigenes was 1,225 bp (Supplementary Table 3A). Most of the unigenes (41,960, 81%) had a length of < 2,000 bp. In general, the number of unigenes decreased with an increase in gene length (Figure 2A).

**TABLE 1:** Sequencing the *P. antidotale* transcriptome in leaf from plants of control (B1, B2, and B3), low salinity (B4, B5, and B6), and high salinity (B7, B8, and B9) treatments.

Sample	Number of clean reads	Size of clean reads (bp)	GC%	Q20 (%)	Q30 (%)
B1	22,864,305	6,859,291,500	55.29	97.82	94.02
B2	32,148,539	9,644,561,700	54.02	98.06	94.57
B3	17,817,291	5,345,187,300	55.38	98.11	94.61
B4	20,525,366	6,157,609,800	55.96	98.17	94.75
B5	24,236,116	7,270,834,800	55.05	98.21	94.84
B6	17,690,930	5,307,279,000	55.05	98.34	95.13
B7	20,029,019	6,008,705,700	55.41	98.08	94.60
B8	19,440,478	5,832,143,400	54.28	98.12	94.64
B9	29,950,686	8,985,205,800	54.01	97.95	94.31

### Annotation and Classification of Unigenes

To provide functional insights, the assembled unigenes were searched against publicly available databases such as NCBI Nr and Nt, SwissProt, GO, KOG, and KEGG pathway using BLASTX algorithm with 10-5 as *E*-value cut-off point. A total of 36,974 (71.3%) unigenes were found for a known protein in at least one of the abovementioned databases. The Nt database presented the highest proportion of annotated unigenes (90.6%), in comparison with the other four databases. The annotated genes were further classified according to differences in their length (Supplementary Table 3B). Approximately 13.3% of identified unigenes were annotated to at least four different databases at the same time, reflecting the accuracy of gene annotation (Figure 2B). Analyses of NCBI Nr (protein) and Nt (nucleotide) database indicated homologs of most identified genes were relatable to those found in other monocots, such as *Sorghum bicolor*, *Zea mays*, *Oryza sativa*, and *Seteria italic* (Supplementary Tables 1, 2).

Gene Ontology enrichment analysis provided information on molecular functions (MF), cellular components (CC), and biological processes (BP) of obtained unigenes in this study. A total of 23,843 unigenes were categorized into 50 functional groups (Figure 2C). The dominant categories for cellular components were cells, cell parts, and organelles. For the biological processes, the annotated genes involved in cellular processes, metabolic processes, and single-organism processes were the most abundant entries. For molecular functions, binding, and catalytic activity were the two most abundant catalogs.

The functions of *P*. *antidotale* unigenes were also predicted by searching the KOG database. In total, 11,528 unigenes were assigned to 25 KOG classification groups (Supplementary Figure 1). Among these, “general function prediction only” accounted for the highest proportion (3,108; 27%), followed by “signal transduction mechanisms” (1,377; 12%), “post-translational modifications, protein turnover, chaperones” (1,280; 11%), and “carbohydrate transport and metabolism” (801; 7%).

To improve our understanding of the biological pathways of identified unigenes, an enrichment analysis with the KEGG pathway was conducted and annotations were assigned. The number of KEGG annotated unigenes (10,602) indicated that “translation” (909; 8.57%), “carbohydrate metabolism” (867; 8.17%), and “folding, sorting, and degradation” (753; 7.1%) were the most abundant biological functions (Figure 2D).

### Identification of Differentially Expressed Genes

**FIGURE 3:**
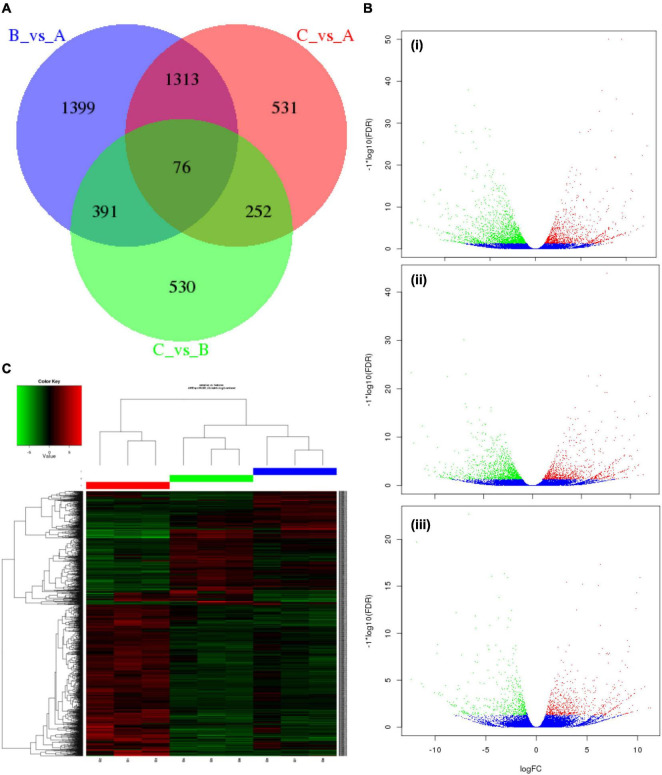
Characteristics of differentially expressed genes (DEGs) in *P. antidotale*. **(A)** Venn diagram showing specific and common salt responsive DEGs among the RNA-seq datasets used in this study: A = control, B = low salinity, C = high salinity. **(B)** Volcano maps representing expression pattern of DEGs (FDR ≤ 0.05 and | log2Ratio| > 1) recorded in treatments: (i) low salinity/control, (ii) high salinity/control, and (iii) high salinity/low salinity. In this study, red spots represent upregulated DEGs, while green spots indicate downregulated DEGs. Those shown in blue are unigenes that did not show obvious changes in salt-stressed plants. **(C)** Heat map representation of DEGs (B1–B3: 0 mM NaCl; B4–B6: 100 mM NaCl, B7–B9: 300 mM NaCl).

To comprehend the transcript expression of *P. antidotale* in response to salt stress, differentially expressed genes (DEGs) in salinity-treated plants were identified upon a comparison against control groups. The correlation analysis to verify consistency among the samples indicated a high similarity among the biological replicates of applied treatments (Figure 2E, r2 > 0.9). The DEGs from low salinity and high salinity treatments were grouped closely in contrast to that of the control treatment. The DEGs were selected if they met the criteria that FDR ≤ 0.05 and a log_2_ fold-change > 1. Interestingly, the number of salt responsive genes was more at low salinity, i.e., 3,179 (1,126 upregulated/2,053 downregulated), when compared with high salinity, i.e., 2,172 (898 upregulated/1,274 downregulated) (Figure 3A and Table 2). The comprehensive results showed that more genes were downregulated than upregulated, under salinity treatment. However, the proportion of upregulated genes was more at high salinity treatment, if compared with that of at low salinity treatment. A heat map, based on FPKM of RNA-seq data, was constructed to profile quantitative differences in the expression levels of DEGs (Figures 3B,C). The expression patterns of DEGs, classified into various groups in the bar according to the log10 (FPKM + 1) value (Du et al., 2010), showed a change from low to high salinity.

**TABLE 2:** The number of upregulated and downregulated DEGs in leaves of *P. antidotale* under different salinity treatments.

DEG Comparison	All DEG	Up-regulated DEG	Down-regulated DEG
Low NaCl vs Control	3,179	1,126	2,053
High NaCl vs Control	2,172	898	1,274
High NaCl vs Low NaCl	1,249	711	538

### Gene Ontology- and Kyoto Encyclopedia of Genes and Genomes Pathway- Enrichment Analysis of Differentially Expressed Genes

The topGO tool (v2.26,^4^) embedded in the R/Bioconductor package was used for enrichment analysis of GO terms, interpretation, and visualization of the results for DEGs (Alexa and Rahnenfuhrer, 2010). Table 3 enlists the most representative GO terms (*P* < 0.05) for each of the GO categories, i.e., BP, MF, and CC. The DNA integration in BPs and the apoplastic region in CCs were the most enriched GO terms in response to both low and high salt whereas the protein serine/threonine kinase activity and RNA dependent DNA polymerase activity were the most representing terms of MFs at 100 and 300 mM NaCl, respectively (Supplementary Figure 2).

**TABLE 3:** GO classification of significantly enriched DEGs by the topGO tool.

Low salinity/Control			
Gene ontology	Number of unigenes in whole transcriptome	Number of unigenes differentially expressed	Corrected *P*-value
DNA integration	541	13	2.06E-06
Protein phosphorylation	1,838	251	4.13E-06
Protein serine/threonine kinase activity	1,349	210	4.20E-06
Integral component of plasma membrane	288	63	2.80E-05
Integral component of membrane	7,174	808	5.08E-05
Apoplast	167	34	5.56E-06
Structural constituent of ribosome	460	16	5.90E-06
Phosphoenolpyruvate carboxylase activity	21	2	8.60E-06
Defense Response	553	102	0.000128

**High salinity/Control**			

**Gene ontology**	**Number of unigenes in whole transcriptome**	**Number of unigenes differentially expressed**	**Corrected *P*-value**

DNA integration	541	5	7.00E-08
RNA directed DNA polymerase activity	327	3	2.50E-06
RNA-dependent DNA biosynthesis	330	3	3.60E-06
Apoplast	167	31	3.80E-06
Structural constituent of ribosome	460	7	6.10E-06
Anchored component of plasma membrane	154	25	1.80E-05
Phosphoenolpyruvate carboxylase activity	21	2	2.50E-05
Extracellular region	474	75	2.90E-05
DNA recombination	597	5	3.10E-05

**High salinity/Low salinity**			

**Gene ontology**	**Number of unigenes in whole transcriptome**	**Number of unigenes differentially expressed**	**Corrected *P*-value**

DNA integration	541	3	4.80E-10
RNA directed DNA polymerase activity	327	4	1.90E-08
RNA dependent DNA biosynthesis	330	4	3.00E-08
DNA recombination	597	8	5.70E-08
Structural constituent of ribosome	460	7	2.20E-07
Phosphoenolpyruvate carboxylase activity	21	2	1.30E-05
Extracellular region	474	26	6.00E-05
Mitochondrial respiratory chain complex I	32	3	0.00014
Eukaryotic translation initiation factor	37	1	0.00163

To further explore significantly regulated biological pathways, a KEGG pathway enrichment analysis was performed. At least, 14 different metabolic pathways were observed to be significantly enriched (*P* ≤ 0.05) in response to NaCl treatments (Table 4). At 100 mM NaCl, DEGs were enriched in “plant hormone signal transduction” and “plant pathogen interaction.” A closer look at their pattern of expression revealed a large proportion of upregulated plant hormone-related genes, i.e., 21 upregulated vs.18 downregulated, while most of the plant pathogen-related genes were downregulated, i.e., 2 upregulated/33 downregulated. At 300 mM NaCl, the DEGs were mainly classified into the “biosynthesis of amino acids” (27 upregulated/2 downregulated). The most represented pathway (*P* ≤ 0.05) in these plants appeared to be “arginine and proline metabolism” (Supplementary Figure 3) when compared with low salinity-treated plants.

**TABLE 4:** Metabolic pathway enrichment analysis of differentially expressed genes (DEGs) as identified by KEGG analyses in leaves of *P. antidotale*.

Low salinity/Control			
Pathway	Gene numbers	*P*-value	Pathway ID
Plant-pathogen interaction	39	0.001035	ko04626
Cysteine and methionine metabolism	24	0.001902	ko00270
Phenylpropanoid biosynthesis	29	0.001902	ko00940
Galactose metabolism	18	0.006195	ko00052
Plant hormone signal transduction	39	0.008158	ko04075
Starch and sucrose metabolism	32	0.009984	ko00500
Arginine and proline metabolism	18	0.021279	ko00330
Fatty acid elongation	11	0.034134	ko00062
Cyanoamino acid metabolism	12	0.040532	ko00460
Phenylalanine metabolism	20	0.046198	ko00360

**High salinity/Control**			

**Pathway**	**Gene numbers**	***P*-value**	**Pathway ID**

Starch and sucrose metabolism	27	0.002004	ko00500
Cysteine and methionine metabolism	18	0.002815	ko00270
Plant-pathogen interaction	26	0.005352	ko04626
Phenylpropanoid biosynthesis	18	0.044869	ko00940
Biosynthesis of amino acids	29	0.044869	ko01230

**High salinity/Low salinity**			

**Pathway**	**Gene numbers**	***P*-value**	**Pathway ID**

Fatty acid elongation	13	7.18E-07	ko00062
Arginine and proline metabolism	14	0.000155	ko00330
Nitrogen metabolism	10	0.00038	ko00910
Cutin, suberin and wax biosynthesis	6	0.021887	ko00073
Glycerophospholipid metabolism	12	0.033996	ko00564

### Single Sequence Repeats Analysis

**FIGURE 4:**
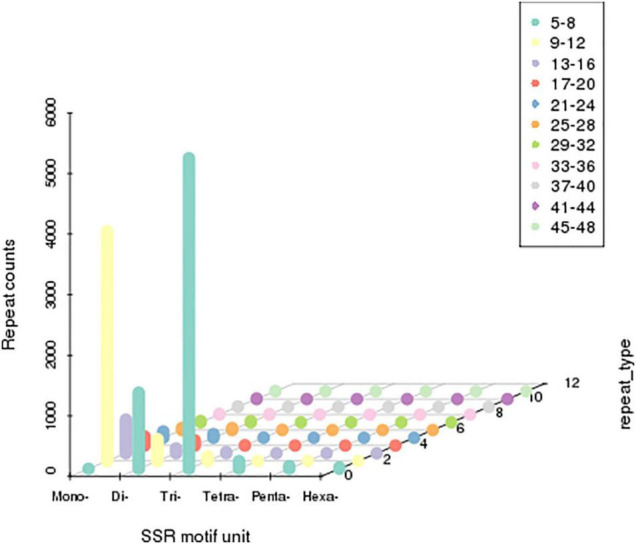
Distribution of simple sequence repeats (SSRs). The X-axis represents the type of SSRs. Z-axis represents the number of SSRs. The numerical value of the Y-axis is coordinate. The number of specific repeats is in accordance with colored illustrations.

*Single sequence repeats* were identified as di- to hexa-nucleotides, consisting of a minimum of four repeats for all motifs. Among the obtained microsatellites, trinucleotide SSRs were the most abundant type followed by di-nucleotide and tetra-nucleotide SSR (Figure 4).

### Proteome Characterization

**FIGURE 5:**
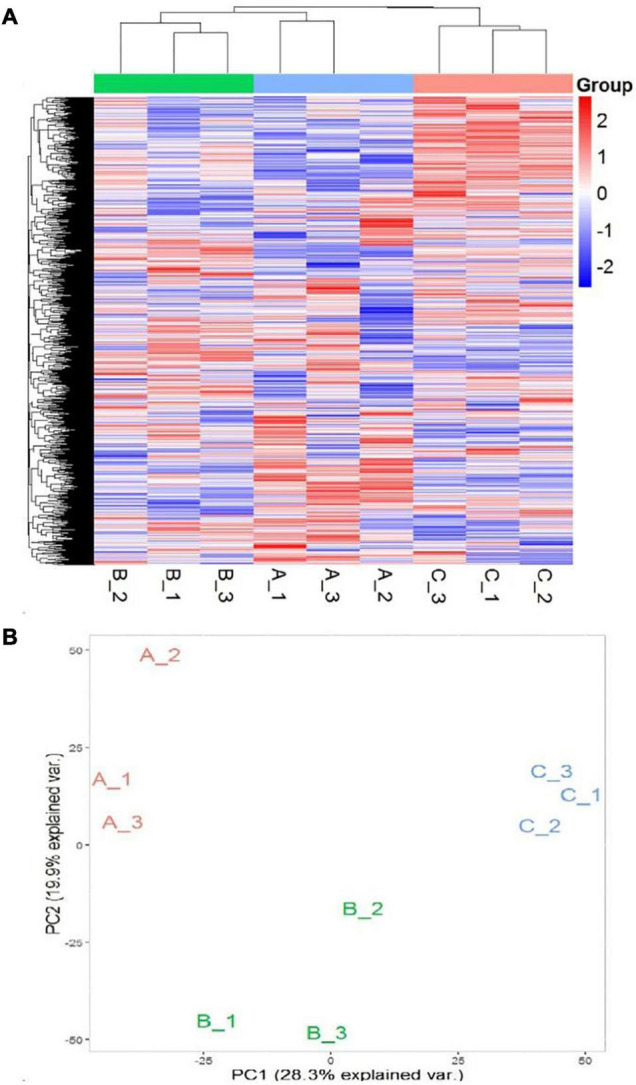
**(A)** Heat map representation of DEPs (A1–A3: 0 mM NaCl; B1-B3: 100 mM NaCl, C1–C3: 300 mM NaCl). **(B)** Principal Component Analysis (PCA) of the proteome data in a 2D graph of PC1 and PC2. The plot shows the treatment effect (loadings) for the control (A1–A3), low (B1–B3), and high (C1–C3) salinity.

**FIGURE 6:**
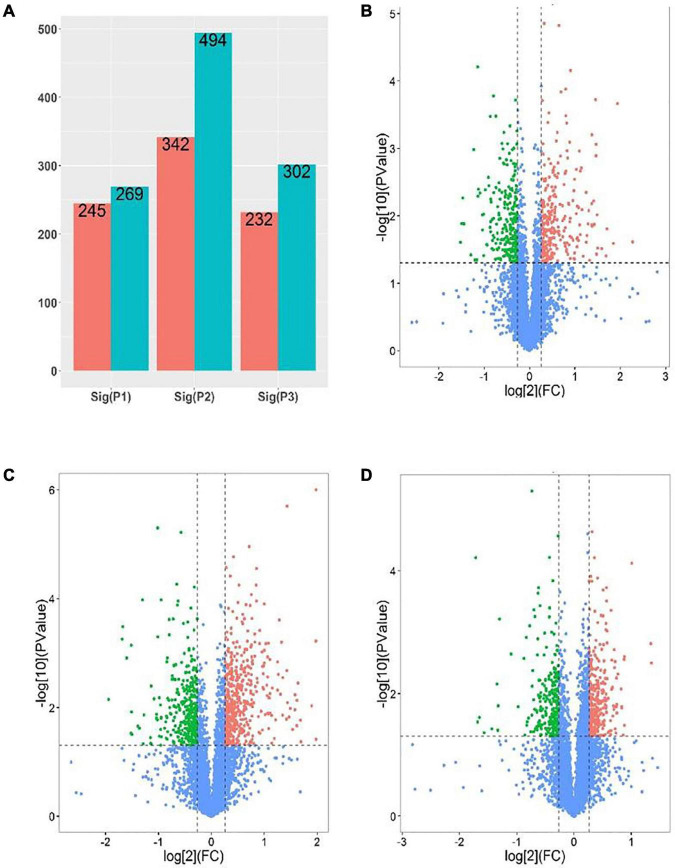
Analysis of DEPs [P1 and panel **(B)**: low salinity/control; P2 and panel **(C)**: high salinity/control; P3 and panel **(D)**: high salinity/low salinity]. **(A)** The number of upregulated (Blue) and downregulated (red) proteins in different comparison groups. **(B–D)** Volcano maps representing expression patterns of DEPs recorded in different comparison groups. Each point represents the difference in protein expression (fold-change) between the two groups plotted against the level of statistical significance. Here, red spots represent upregulated DEGs, while green spots indicate downregulated proteins. Those shown in blue are proteins that did not show obvious changes in compared groups.

In order to further develop our understanding of salt tolerance mechanisms of *P. antidotale*, proteomes in response to applied treatments were also analyzed. A total of 5,148 non-redundant proteins were produced, based on 26,587 unique peptides (Supplementary Table 4A). Among these, 4,983 proteins were common to the three datasets. The variation in protein expression in response to salinity treatments is shown (Figure 5A). Principal component analysis (PCA) represented a closer association of biological replicates rather than salinity treatments. PC1, accounting for 28.3% of the total variation, distinctly separated plants of low salinity treatment to that of high salinity and control treatment, while PC2, explaining 19.9% of the total variation, indicated differences between control and high salinity treatment (Figure 5B). The comparison of salinity-treated plants with control revealed differential expression of 514 (269 upregulated/245 downregulated) and 836 (494 upregulated/392 downregulated) proteins (DEPs) at low and high NaCl, respectively. When compared with low salinity, 302 upregulated and 232 downregulated proteins were recorded at high salinity (Figure 6 and Supplementary Table 4B).

### Functional Annotation of Differentially Expressed Proteins

**FIGURE 7:**
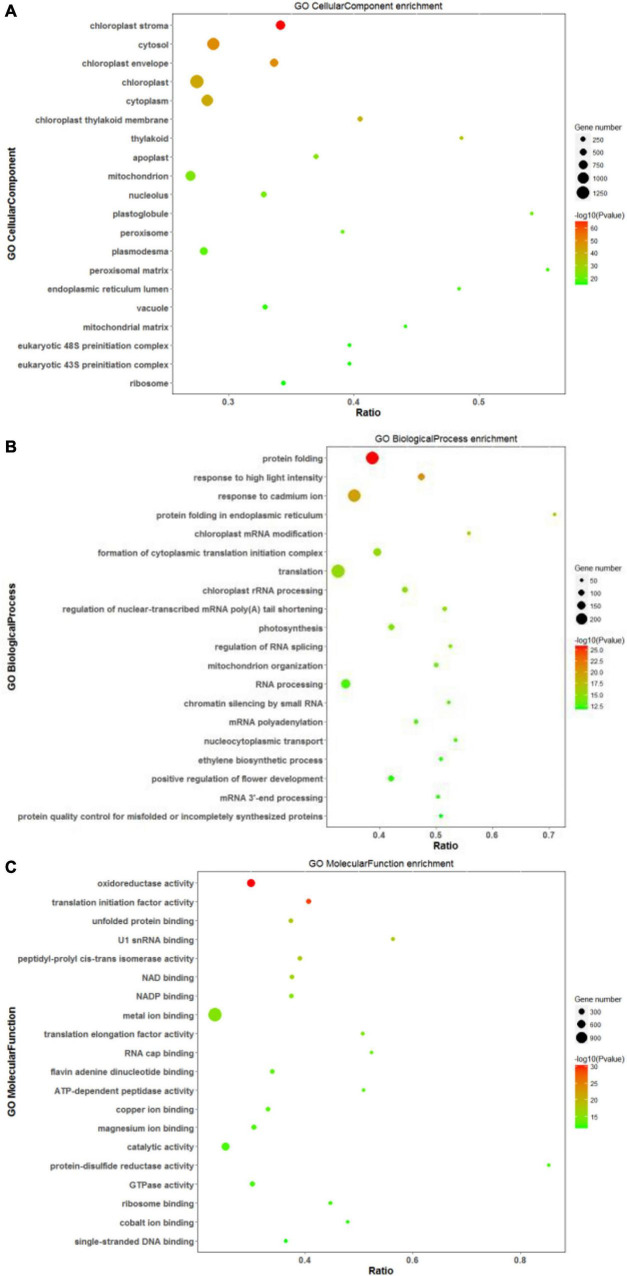
Scatter diagram of gene ontology (GO) classification for DEPs. In this scatter diagram, the top 20 GO terms in the cellular component **(A)**, biological process **(B)**, and molecular function **(C)** categories are presented. The X-axis corresponds to the ratio (DEPs in this term to all the proteins in this term), and the Y-axis represents a different term. The magnitude of the dots displays the number of proteins, and the q-value is described by the color classification.

Based on annotations from the Uniprot database, differentially expressed proteins were assigned functional categories to GO and KEGG terms. As shown in Figure 7A, the significantly enriched GO terms in the CC category included “chloroplast: stroma and envelope,” “cytosol,” and “cytoplasm.” Compared with other treatments, DEPs related mainly to chloroplast and its components (stroma and envelope) were downregulated at high salinity, while those related to “endoplasmic reticulum” were upregulated. DEPs assigned to “ribosomes” were primarily up-regulated in response to saline treatments (Supplementary Table 5). In the BP category, “protein folding,” “translation,” and “response to high light intensity” were the most significantly enriched terms (Figure 7B). Out of these DEPs, translation-related proteins were upregulated at both 100 and 300 mM NaCl. In contrast, to control treatment, downregulated DEPs at 100 and 300 mM NaCl were predominantly involved in “homeostasis of meristem” and “sucrose biosynthesis,” respectively. The comparison of high salinity responsive proteome with that of low salinity indicated upregulation of proteins related to “oxidative stress response” and “hydrogen peroxide catabolism” while “chloroplast organization” related proteins were downregulated (Supplementary Table 6). For the MF category, “oxidoreductase activity,” “translation initiation factor activity,” “metal ion binding,” and “catalytic activity” were the most significant terms (Figure 7C). When compared with control, DEPs related to the “structural constituent of ribosome” were upregulated, while those associated with the “structural constituent of cell wall” were downregulated. The comparison of high salinity with low salinity indicated upregulation of “peroxidase” related proteins and downregulation of “ligase” linked proteins (Supplementary Table 7).

**FIGURE 8:**
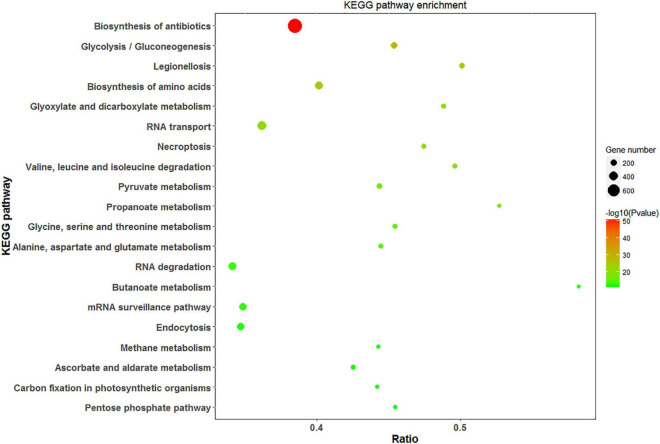
Scatter diagram of KEGG pathway enrichment for DEPs. The top 20 pathways were listed in the plots. The X-axis corresponds to the ratio of DEPs in this pathway to all the genes in this pathway, and the Y-axis represents a different pathway. The magnitude of the dots displays gene numbers, and the q-value is described by the color classification.

Analysis of DEPs with the KEGG pathway revealed their primary participation in the biosynthesis of antibiotics and amino acids, Glyoxylate and dicarboxylate metabolism, and RNA transport (Figure 8). In response to saline treatments, the main KEGG terms for upregulated proteins were “ribosome,” “biosynthesis of phenylpropanoid,” and “protein processing in endoplasmic reticulum.” “Glutathione metabolism,” “pentose phosphate pathway,” and “ascorbate and aldarate metabolism” were among the most significantly upregulated pathways at high salinity in contrast to low salinity. DEPs contributing to the “mitogen-activated protein kinase (MAPK) signaling pathway” and “carbon fixation” were significantly downregulated at 100 mM and 300 mM NaCl, respectively. In addition to carbon fixation, “porphyrin and chlorophyll metabolism” was downregulated at high salinity when compared with low salinity (Supplementary Figure 4).

### Protein-Protein Interaction Analysis

**FIGURE 9:**
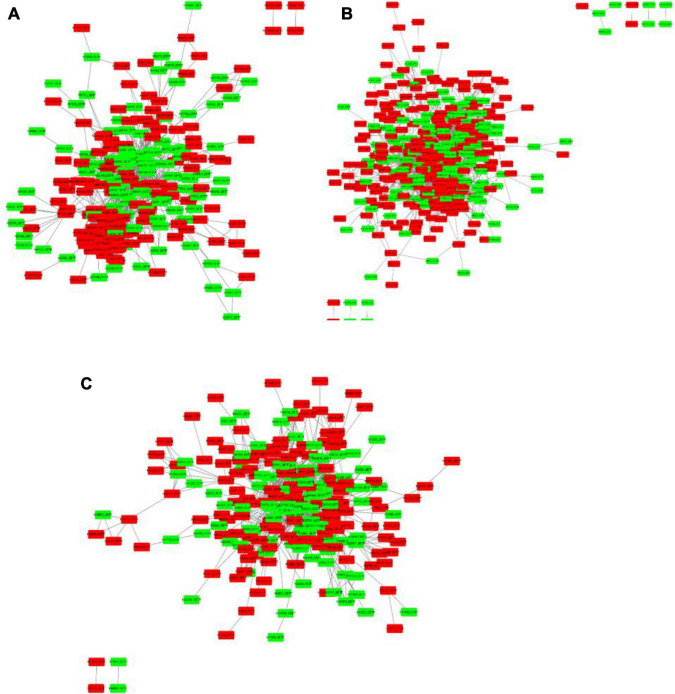
Protein-protein interaction (PPI) analysis of the DEPs in *P. antidotale* exposed to salt treatments: **(A)** low salinity/control, **(B)** high salinity/control, and **(C)** high salinity/low salinity. The boxes represent proteins while the straight lines represent the interactions between different proteins. Red boxes indicate the upregulated DEPs and green arrows indicate the downregulated DEPs.

To get functional links for DEPs, a regulatory network for up and downregulated proteins was built using STRING analysis. Among the 1,884 DEPs, three regulatory networks of the DAPs containing 228, 399, and 231 nodes (low salinity vs. control, high salinity vs. control, and high salinity vs. low salinity, respectively) were obtained. There was considerable overlapping among the major clusters, particularly for DEPs involved in carbohydrate metabolism, ribosome structure, translation, and transportation. Ribosomal subunits were the most important protein up-regulation hubs in response to salinity treatments, while MAPK and RuBisCo small chain were the most important protein downregulation hubs at 100 and 300 mM NaCl, respectively, in the constructed networks. In contrast to 100 mM NaCl, proteins involved in the biosynthesis of chlorophyll and fatty acids, and carbohydrate metabolism constituted the downregulated protein hubs while those involved in mitochondrial electron transport, oxidative pentose phosphate pathway, and thioredoxin were the protein-upregulation hubs at 300 mM NaCl. These proteins had multiple interactions and constituted a complex network in response to salt stress (Figure 9).

## Discussion

Increasing saline lands has made it critical to analyze the salt stress responses of halophytes with the aim to develop salt-tolerant crops through molecular breeding and transgenic approaches. Identification of specific salt responsive genes based on either transcriptome or proteome analysis has been conducted in several plants. However, there is a lack of research on conjoint analyses of transcriptome and proteome for any of the halophytes. Since a weak correlation existed between the transcriptome and proteome profiles, comprehensive studies including both transcriptome and proteome tools had provided researchers an opportunity to identify a relatively large pool of stress-related genes and proteins constituting salt tolerance strategies. *P. antidotale*, a C4 halophytic grass, has been deeply investigated for its underlying physiological and biochemical aspects of salt tolerance. The current study discusses the role of several genes involved in salt response pathways of *Panicum* with emphasis on their expression at both transcriptional and translational stages.

Exposure to saline conditions triggers a series of responses in plants, including signal transduction, translational modifications, ion trafficking, energy, and carbohydrate metabolism (López-Marqués et al., 2020: Guo et al., 2021; Mann et al., 2021). While halophytes grow optimally at low salt concentrations, high concentrations of salts negatively affected the biological processes ranging from plant water uptake to antioxidant defense mechanisms. Considering the variability of plant responses to the severity of stress conditions, plants are grown under 0 (control), 100 (low), and 300 mM (high) salinity treatments were used to identify metabolic shifts probably related to their salinity tolerance. Results indicated the prevalence of weak correlations between transcripts and proteins. For instance, most of the differentially expressed genes (DEGs) are downregulated while a large proportion of differentially expressed proteins (DEPs) are upregulated under salinity. Many of the significantly enriched GO and KEGG terms for DEGs and DEPs were consistently suggesting the similarity among the general response of plants under salt treatments. However, some differences were also encountered. For instance, terms like “flavonoids biosynthesis,” “N-Glycan biosynthesis,” “Glucosinolate biosynthesis,” “cutin, suberin, and wax biosynthesis,” and “lysosomes” were only enriched under high salinity, while others such as “oxidative stress” and “biosynthesis of amino acids” were significantly up-regulated when compared with low salinity treatment. These processes may be responsible for compromised plant growth and survival under high saline conditions. Differently, “transmembrane transport” and “protein phosphorylation” were enriched and the “phenylpropanoid pathway” was upregulated at low salinity. Conjoint analyses (transcriptome and proteome) revealed that carbohydrate metabolism was the most significantly affected process at high salinity. Thus, some novel insights for the salt tolerance mechanism of *P. antidotale* under low and high concentrations of NaCl were obtained and a putative model for plant salt tolerance was constructed.

### Carbon Metabolism and Bioenergy

Our present study indicated immense alterations in plant photosynthetic and carbon fixing machinery. The upregulated expression of the oxygen-evolving complex of PSII (OEC) reflected a greater efficiency of the oxygenic photosynthetic apparatus under saline (low and high) conditions. However, high salinity induced damage to photochemical reactions and energy synthesis was evident with downregulated DEGs and DEPs constituting PSII (D1, D2, and cp43), PSI (PsaJ), photosynthetic electron transport (fd, FNR), and ATP-synthase complex (beta and alpha subunits). The decreased protein abundance of a light-harvesting chlorophyll protein complex (LHC) mirrored disturbance in light quenching capacity (Farhat et al., 2021), and therefore, the exposure of high salinity treated plants to excessive light energy (Lin et al., 2018). Accordingly, GO analysis for DEGs revealed “response to high light intensity” as an exclusively enriched term in these plants as recorded for another halophytic grass (Mann et al., 2021). Although the transcript expression of the enzyme beta-carotene 3-hydroxylase, which regulates biosynthesis of carotenoids (β-carotene and zeaxanthin), was increased; the pattern of its protein expression was not consistent. Moreover, the downregulated protein abundance of enzymes participating in the xanthophyll cycle (violaxanthin de-epoxidase and zeaxanthin epoxidase) triggered photoinhibition and photooxidative damage. Thus, the high salinity treatment significantly damaged photochemical reactions and photosynthetic machinery of *P. antidotale*, reinforcing its disturbed photosynthetic performance as reported in our published eco-physiological study (Hussain et al., 2015, 2020).

Our study revealed the consistent increase in transcripts and proteins of phosphoenolpyruvate carboxylase (PEPC) under saline treatments, suggesting the powerful role of this gene in salt tolerance and utilizing it as a molecular biomarker to investigate salt response in future research. The overexpression of C4 PEPC has been declared a promising approach to improve abiotic stress tolerance of several plants including C3 crops (Shen et al., 2015; Kandoi et al., 2016). However, the impairment in subsequent events of C-fixation, i.e., generation of pyruvate and release of CO_2_ in bundle sheath cells, at high salinity was suggested with downregulated protein expression of malate dehydrogenases and alanine transaminase. The damage to C-fixing ability was further evident with decreased expression, at least at the protein level, of Calvin cycle enzymes such as RuBisCo, glyceraldehyde-3-phosphate dehydrogenase, phosphoribulokinase, and ribose 5-phosphate isomerase (RuBP). Although increased transcripts for Rubisco, RuBP, and transketolase represented the adaptive strategy of plants to survive at low salinity, no significant change in their protein abundance was recorded. In line with the above, the possibility of low salinity-induced modulation of the photosynthetic carbon fixation could be ruled out. The optimal performance of *P. antidotale* in terms of photosynthetic capacity at low salinity was consistent in published results (Koyro et al., 2013; Hussain et al., 2015, 2020: Rasouli et al., 2021).

### Sucrose and Starch Metabolism

This study indicated decreased transcripts for beta-fructofuranosidase (Invertase), but its protein abundance increased specifically at 300 mM NaCl, suggesting the elevated levels of hexose moieties in these plants. The enhanced soluble sugars contributed to increasing water uptake under saline environments by lowering the solute potential of the cells (Acosta-Motos et al., 2017). We also noticed an increased abundance of enzymes catalyzing starch biosynthesis, i.e., glucose-1-phosphate adenylyltransferase, starch synthase, 1,4-alpha-glucan, amylases, and glycogen phosphorylase, in response to saline treatments. Hence, the increased starch acts as osmotic carbohydrates that appear an effective approach of plants to store some fraction of photosynthetically assimilated carbon as an energy reservoir (Thalmann and Santelia, 2017). The upregulated abundance (transcripts and proteins) of enzymes catalyzing the trehalose production, i.e., trehalose 6-phosphate synthase and trehalose 6-phosphate phosphatase reflect its important role as a stress protectant (Lin et al., 2019) in high salinity treated *P. antidotale*. Combining these findings illustrated the adaptive strategy of our test species i.e., the accumulation of sugars as compatible solutes, to thrive salinity-induced physiological drought.

### Respiration

Increased respiratory activity under saline conditions illustrates the increased allocation of stored carbon reserves for salt tolerance mechanisms (Jacoby et al., 2011). The current study demonstrated that plant metabolism was inhibited, i.e., the decreased protein abundance for many enzymes related to glycolysis at 300 mM NaCl, although their transcripts increased under salinity (low and high). Low salinity inhibited the enzymes of the oxidative phase of the pentose phosphate pathway (PPP), while the abundance of enzymes for the non-oxidative phase was up-regulated, suggesting active nucleotide metabolism. Interestingly, the enzymes of the TCA cycle were increased under saline (low and high) treatments reflecting the increased energetic demands to endure saline conditions. However, the sustained respiratory rates could not be claimed for high salinity treated plants as the enzymes catalyzing the splitting of glucose into pyruvate, i.e., the steps of glycolysis decreased in their abundance. The efficiency to produce ATP was significantly affected due to damage in several components of the mitochondrial electron transport chain, i.e., NADH dehydrogenase, and ATP synthase. Whereas increased ATP production in low salinity plants was displayed with increased expression of inorganic pyrophosphate and H^+^ transporting ATPase although the expression of remaining components of respiratory ETC complexes remained unaffected. Overall, these results suggested efficient respiration at low salinity but disturbed respiration in high salinity treated plants.

### Amino Acid Metabolism

Metabolic regulations enable plants to survive stressful environments. Accordingly, a significant proportion of DEGs at high salinity was related to amino acid metabolism. Increased transcripts expression for enzymes related to several amino acids i.e., methionine, proline, valine, leucine, isoleucine, and glutamine indicated their role in osmotic adjustments under salt stress. In addition, amino acids participate directly and/or indirectly as antioxidants (Maghsoudi et al., 2018). For instance, enhanced expression of transcript and protein of methionine metabolism controls the synthesis of glutathione and regulates methylation events which are essential for cellular signaling in salinity treated *Panicum*. It must be noted, however, that enzymes regulating proline metabolism did not increase and those regulating metabolism of leucine, valine, and isoleucine, were even downregulated. In contrast, the exposure of plants to low salinity treatment increased the expression of enzymes regulating proline biosynthesis such as glutamate-5-semialdehyde dehydrogenase/P5CS, glutamate 5-kinase, pyrroline-5-carboxylate reductase, and 1-pyrroline-5-carboxylate dehydrogenase transcripts and/or proteins. It is interesting as the antioxidant ability of proline by being an oxygen quencher under stress has been acknowledged (Szabados and Savouré, 2010). In particular, the considerably increased expression of P5CS transcript (>8-folds) reflected it as an important candidate to understand the processes of abiotic stress tolerance in several plants (Maghsoudi et al., 2018; Anton et al., 2020). Together these results suggest that better performance of *P. antidotale* to low salinity was related to consistently up-regulated metabolism (transcripts and proteins) for key amino acids i.e., proline and methionine; the high salinity treatment altered the functional elucidation (protein abundance) of upregulated transcripts of various amino acids, but lysine and arginine mainly contributed to the osmotic tolerance of these plants.

### Reactive Oxygen Species Scavenging and Defense Mechanisms

High salinity treated plants experienced oxidative damage owing to the prolonged presence of intracellular ROS and, thus, a disturbed equilibrium between detoxification and the generation of H_2_O_2_. Although enzymes such as superoxide dismutase (SOD) and ascorbate peroxide (APX) increased at high salinity, which is consistent with their increased activity as reported previously (Hussain et al., 2015), the undetected or deceased expression of other antioxidative enzymes, i.e., GPOX, MDHAR, GR, and GST, led to an oxidative environment which was also reflected with increased (>4-folds) expression of cell-wall specific ascorbate oxidase (AO) genes. In contrast, the up-regulated abundance of low H_2_O_2_ affinity catalase (CAT) in low salinity treated plants allowed us to consider the signaling role of H_2_O_2_ in various cellular processes like cell cycle, growth, and development (Sofo et al., 2015). The downregulated expression of AO in these plants also affirmed the presence of a reduced environment in the apoplast and has been related to the ability of plants like tobacco and *Arabidopsis* to survive saline conditions (Fotopoulos et al., 2006). This differential contribution of antioxidant machinery to mitigate oxidative stress at low and high salinities was consistent with reports for other halophytes (Asrar et al., 2017).

### Signaling Pathways

Saline treatments caused a disturbance in various signaling cascades and, therefore, affected various developmental processes of *P. antidotale*. For instance, several elements composing the three tiers of MAPK signaling cascade, e.g., MEKK2, MLKT, and MEK, were downregulated. The increased abundance of PP5 and dual-specificity protein phosphatases (MKP) further reflected inhibited activity of MEKK2 and extracellular signal-related kinase (ERK). The activation of the MAPK signaling pathway is observed to regulate numerous transcription factors with a role in salt resistance (Wang et al., 2014). However, the obtained weak correlations among ERK transcripts (downregulated) and protein (unchanged) signifies the analysis of plant responses at a multi-omics level to fully validate the salt tolerance mechanisms. The expression of key molecules involved in the PI3K-Akt pathway—an intracellular signaling pathway to promote metabolism, cell cycle, growth, and apoptosis in response to extracellular signaling, was influenced under saline conditions. The downregulated expression of Rac1 protein suggested the inactivation of PI3K class Ia proteins whereas an upregulated expression of guanine nucleotide-binding protein reflected the active status of PI3K class Ib proteins, suggesting altered recruitment of protein kinase B (AKT) at the plasma membrane. The damage to this signaling cascade was more evident at high salinity, i.e., increased PTEN and PP2A, and decreased PDK1. Accordingly, downstream target proteins of AKT, i.e., glycogen synthase kinase (GSK3) and cyclin-dependent kinase (CDK) were decreased in these plants reflecting the toxic effects of high salinity on glycolysis/glucogenesis and cell cycle.

The components of the Ca^2+^/SOS signaling cascade were relatively more inhibited by high salinity, reflecting the inability of these plants to control ion homeostasis as described (Roy et al., 2014: Dindas et al., 2021). Although the increased abundance of phospholipase pathway (i.e., PLCδ) suggested enhanced cytoplasmic Ca^2+^ due to its release from the endoplasmic reticulum, the reduced expression of calmodulin (CaM), an intermediate sensor of free intracellular Ca^2+^, therefore, indicate a functional SOS pathway in salt-treated *P. antidotale*. The upregulated transcripts (at 300 mM NaCl) and proteins (at 100 and 300 mM NaCl) for voltage-dependent anion channel protein (VDAC) of the outer mitochondrial membrane may be associated with increased transport of cytoplasmic calcium into mitochondria, i.e., a phenomenon proposed to achieve mitochondrial calcium homeostasis as a plant response to abiotic stress (Zhang et al., 2015; Carraretto et al., 2016). The relatively greater reduction in the abundance of a serine/threonine protein phosphatase (CaN) and 14-3-3 proteins, i.e., the inhibitors of the SOS pathway, was related to better survival of low salinity treated plants as discussed (Zhou et al., 2014; Tan et al., 2016).

### Cell Cycle

The compromised plant growth under (high) saline conditions was mainly due to increased accumulation of cyclin A, and inhibited expression of cyclin-dependent kinases, and proliferating cell nuclear antigen.

### RNA Processing and mRNA Surveillance Pathways

Exposure to high salinity enhanced the abundance of Ran-GTP, NMD3, and many components of the nuclear pore complex, indicating increased transport of RNAs. In contrast, the increased abundance of exportin at low salinity suggests specific nucleocytosolic export of tRNA which is relatable to their protein synthesis machinery. High salinity also altered the processing of mRNA such as decreased the abundance of 3′ poly(A) binding proteins (PABPs) and RNA-binding protein Musashi (MSI), which give stability to the nuclear-exported mRNA and increased abundance of mRNA-decapping protein and ATP-dependent RNA helicase.

Environmental constraints such as salinity, are often prone to produce aberrant mRNAs, i.e., with premature termination codon (PTC), and therefore alter transcriptome and proteome response of the plant cell (Roundtree et al., 2017). The increased abundance of UP-Frame shift proteins, several components of Exon Junction Complex (EJC), and 5′-3′ exoribonuclease (XRN2), particularly in low salinity treated plants, reflects active degradation of defected mRNA by surveillance pathways as explained by Boehm and Gehring (2016). The degradation of faulted mRNA in 3′ to 5′ direction (Fan et al., 2017) was evident at high salinity as indicated with up-regulated exosome coactivator complex components i.e., ski complex (ski8) and TRAMP complex (Mtr4) but inhibited abundance of XRN2 allowed us to speculate about the accumulation of aberrant mRNAs in these plants.

### Protein Synthesis, Export, and Processing

Low salinity hampered protein biosynthesis, such as the expression of methionyl-tRNA synthetase, i.e., the first enzyme to initiate the translation, and synthesis of ribosomal components, i.e., large and small subunits, was downregulated. Though, damage to the protein factory cannot be claimed as their protein abundance remained unchanged or even increased, thus, indicating a weak correlation between transcript and proteome analyses. On the other hand, high salinity treatment increased the abundance of ribosomal subunits and multiple factors involved in ribosomal assembly i.e., UTP and MPP10 complexes, and rRNA modification i.e., nucleolar proteins (1, 56, 58), ribonucleoprotein complexes (DKC, NHP), suggesting enhanced protein biosynthesis or repair of salt-stressed proteins to cope with saline environments (Liu et al., 2019). However, the localization of newly synthesized proteins was altered due to inhibited expression of signal recognition particle (SRP) and its receptors in these plants. Interestingly, the processes such as glycosylation, folding of nascent polypeptides, and transport of secretory proteins from ER to the Golgi complex were increased as reflected with increased SAR1, alpha-1,3-glucosidase, calreticulin, calnexin, ER luminal chaperones (NEF, BiP, heat shock protein 90 kDa beta). Moreover, the increased abundance of protein disulfide isomerase (PDI) suggested active hydrolyzation of disulfide bonds, particularly in terminally misfolded proteins for their subsequent breakdown *via* the ER-associated degradation (ERAD) pathway. Degradation of damaged proteins was also evident with upregulated expression of multiple components of ubiquitin-mediated proteolysis, i.e., E1 (ubiquitin-activating enzyme), E2 (ubiquitin-conjugating enzyme), E3 (ubiquitin ligase), and other ERAD factors (HSPs, U-box containing protein, and S-phase kinase-associated protein). The expression of an ER stress sensor i.e., serine/threonine-protein kinase/endoribonuclease inositol-requiring enzyme (IRE1), was downregulated, which limits unfolded protein response (UPR) and damages plant adaptability to stress environments (Halbleib et al., 2017). Thus, the asynchrony among the levels of targeted protein degradation and that of ER stress sensors may define the inability of *P. antidotale* to perform optimally at high salt regimes.

### Plant Hormone Signal Transduction

The damage to phytohormones signaling and discrepancy in their levels as revealed by the transcripts and proteins component and was more pronounced at high salinity. Numerous auxin response factors were downregulated in response to applied treatments. High salinity induced inhibition in Arabidopsis histidine phosphotransfer protein (AHP) and subsequent B-Arabidopsis response factors (B-ARR) reflects less development of shoots as reported previously (Xie et al., 2018) even though, the levels of cytokinin receptor were high in these plants. We noticed an increased abundance of nuclear gibberellin receptor (GID1), which binds with DELLA proteins i.e., the repressor of GA signaling, for their subsequent degradation *via* 26S proteasome complex, but the decreased expression of 26S proteasome, RING-type E3 enzyme, and F-box protein of SCF complex, at 300 mM NaCl, allowed us to presume the accumulation of DELLA proteins and, therefore, disturbing plant growth and development (Itoh et al., 2008). The enhanced transcript and protein abundance of ABA-responsive transcription factor, ABA receptor (PYR/PYL), 2C Ser/Thr protein phosphatases (PP2C), specifically at high salinity, suggested ABA-mediated mechanisms such as stomatal closure in these plants (Shabala et al., 2012; Huang et al., 2019). Moreover, the downregulated abundance of ethylene receptor and ethylene insensitive protein (EIN2) in high salinity treated plants was related to inhibited downstream signaling. Impairments in salicylic acid-mediated signaling pathways in response to low and high salinity were evident with decreased abundance of i.e., regulatory protein (NPR1), transcription factor (TGA), and pathogenesis-related protein 1 (PR1). We recorded inhibited expression of several components of the jasmonic acid signaling pathway, such as jasmonate ZIM domain-containing protein (JAZ), coronatine-insensitive protein (COI1), Brassinazole resistant (BZR) gene, and BR-receptor (BRI1). However, the decreased abundance of brassinosteroid insensitive protein (BIN2)—a negative regulator of the BIR1 pathway, and enhanced xyloglucosyl transferase (TCH4) with function in cell elongation, under saline conditions, reflects strategies of our test species to resist saline conditions.

### Lipid Metabolism

Saline conditions boosted the abundance of palmitoyl-protein thioesterase (PPT), which mediates acylation and diacylation of proteins like Ras-family small GTPases and G proteins and enable their signaling function at the plasma membrane (Rocks et al., 2010). We noticed that low salinity treatment inhibited elongation of fatty acyl chains with length < 16 carbons by reducing abundance of enoyl- (acyl-carrier protein) reductase, whereas high salinity inhibited extension of fatty acyl chains of length > 16 carbons by downregulating enzymes like 3-ketoacyl-CoA synthase and 17 beta-estradiol 17-dehydrogenase. The application of 300 mM NaCl further enhanced the synthesis of saturated fatty acids, i.e., elevated levels of fatty acyl-ACP thioesterase type B (FATB). The increase in the proportion of saturated fatty acids alters nutrient transport and signaling activities (Mansour, 2013) as reported for various plants (Wu et al., 2005; Tsydendambaev et al., 2013).

The adverse effects of high salinity on membrane integrity and function were reflected with the downregulated abundance of acetyl-CoA carboxylase, i.e., the rate-limiting enzyme of fatty acid biosynthesis, and other enzymes such as acyl-ACP reductase. Although the expression of long-chain acyl-CoA synthetase increased in these plants, the reduced levels of enoyl-acyl carrier protein reductase I (fabI), and up-regulated abundance of acyl-CoA oxidase, indicated the involvement of acyl-CoA synthetase in the degradation of synthesized fatty acids *via*β-oxidation rather than its function in the biosynthesis of lipids (e.g., triacylglycerols, phospholipids, etc.). Accordingly, the enzymes of the beta-oxidation pathway, i.e., acyl-CoA hydratase and acyl-CoA dehydrogenase, increased in their abundance. The breakdown of fatty acids *via* peroxisome and mitochondria is beneficial under stress as it releases energy and carbon skeletons to be utilized in other metabolism but is generates cytotoxic ROS, thereby, causing plants to experience oxidative stress.

Although the key enzymes of wax biosynthesis decreased, aldehyde decarbonylase and alcohol-forming fatty acyl-CoA reductase, showed enhanced expression in response to salinity. while the enzymes involved in the synthesis of cutin monomers (i.e., ω-hydroxy, and α,ω-dicarboxylic fatty acids), were specifically up-regulated under hypersaline conditions which support its part to restrict non-stomatal water loss *via* cuticle. We recorded a decrease in expression of omega-hydroxypalmitate *O*-feruloyl transferase with a function in increasing the durability of the cuticle by accumulating feruloyloxypalmitic acids and in the biosynthesis of suberin i.e., a glycerolipid polymer located primarily in internal cell walls (Molina et al., 2009). Our findings contrast with other studies where the accumulation of suberin was implicated in plant stress tolerance by serving as an apoplastic barrier to salts (Krishnamurthy et al., 2009; Zhang et al., 2020).

### Secondary Metabolism

The better tolerance of *P. antidotale* to low salinity was evident with increased lignification, i.e., caffeoyl-CoA *O*-methyltransferase and enzymes catalyzing the synthesis of lariciresinol and secoisolariciresinol. Moreover, the enzymes involved in interconversions of alcohols to aldehydes/ketones increased in these plants. The produced lipophilic metabolites, such as 2-Naphthalenemethanol, have been shown to improve stress detoxification and tolerance (Soliman et al., 2019). Though the transcript expression of caffeoyl-CoA *O*-methyltransferase and other enzymes which have a role in lignin polymerization, i.e., *S*-adenosylmethionine and *trans*-cinnamate 4-monooxygenases, increased at high salinity, their protein contents reflected a weak correlation. Emerging evidence suggests that lignin biosynthesis is partly due to the culmination of chalcone accumulation (Díaz-Tielas et al., 2016; Eloy et al., 2017) and, thus, the relatively high expression of chalcone synthase relates well with disturbed lignification at high salinity. In contrast, the relatively more flavonoids facilitated these plants to resist high salt stress, i.e., enzymes involved in the synthesis of various flavonoids, i.e., flavans (Catechin, Epicatechin, Epigallocatechin), flavones (Luteolin, Baicalein), flavonols (Kaempferol, Myricetin, Quercetin), and anthocyanidins (Pelargonidin, Peonidin, Petunidin), with their suggested roles in antioxidant activity and metal chelation (Mathesius, 2018). However, the expression of mono-, di-, or tri- terpenes biosynthesis-related genes, i.e., neomenthol dehydrogenase, ent-kaurene oxidase, gibberellin-44 dioxygenase, NAD^+^-dependent farnesol dehydrogenase, was severely repressed at 300 mM NaCl, suggesting impairments in accumulation of phytohormones, such as the sesquiterpenoid ABA and the diterpenoid gibberellic acid (GA) (Han-Chen et al., 2020). On the other hand, the low levels of flavonoids coupled with increased biosynthesis of terpenes reflected the adjustment in secondary metabolism to withstand a low salinity environment.

## Conclusion

This study reported and analyzed the salt tolerance mechanisms of *P. antidotale* at low and high salinity by using a comparative transcriptome and proteome approach. The results revealed a large number of DEG and DEP indicating the differential regulation of salt-responsive genes to direct plant responses. Genes contributing either to the structure or the functioning of photosynthetic machinery showed inhibited transcription under both treatments, but their protein expression decreased only by high salinity. The respiratory process was decreased at high salinity and therefore, could not sustain the higher energetic demands of the plants. Though the components of various cellular metabolisms were disturbed at low salinity, the treated plants showed better growth due to the upregulation of several salt-resistant genes, such as proline biosynthesis, antioxidative defense machinery, Ca^2+^/SOS signaling cascade, cell growth, mRNA surveillance pathways, and secondary metabolism. In contrast, a greater proportion of salt-resistant mechanisms were affected at high salinity. The transcription of amino acid metabolism-related genes increased in these plants, but their protein abundance did not change accordingly. In contrast, the accumulation of sugars and trehalose played a role in protecting against osmotic stress and physiological drought. Numerous genes related to the cell cycle and gene expression were downregulated. Moreover, genes involved in DNA repairing and the processing of aberrant mRNA exhibited a decrease in abundance. The increased translation as demonstrated by the increased abundance of related machinery and enzymes was, therefore, not an advantage. The growth of plants was further restricted as a disturbance in fatty acid metabolism, i.e., stimulated degradation of fatty acids and a high proportion of saturated fatty acids, altered the integrity of cell membranes. The expression of genes involved in lignan metabolism also decreased at least at protein levels. ABA-mediated downstream signaling was more pronounced in these plants, which suggests the role of physiological mechanisms like stomatal closure in limiting biomass accumulation (Hedrich and Shabala, 2018). In addition, the increased expression of numerous inhibitors of the SOS signaling cascade suggested impairments in stress-activated responses. Even though, the expression of calreticulin, calnexin, ER luminal chaperones, and heat shock proteins was increased in these plants, the incomplete operation of the antioxidant defense system did not scavenge excessive ROS and, therefore, suggests a high salinity induced oxidative damage. Besides, these data provide numerous valuable candidate genes with an identical pattern of expression at transcript and protein levels that can be utilized to screen the salt tolerance of other halophytes. In addition, the obtained information has scope to transfer the character of salt tolerance to non-halophytes. Overall, the findings of this study contributed to understanding the molecular basis of optimal and restricted plant growth at low and high salinity, respectively.

## Data Availability Statement

The original contributions presented in the study are publicly available. This data can be found here: National Center for Biotechnology Information (NCBI) BioProject database under accession number PRJNA757558.

## Author Contributions

TH and XL conceived the idea. TH and WZ performed the experiments. HA, TH, and WZ analyzed the data. TH and HA wrote the manuscript. BG and XL corrected the manuscript. All the authors approved the final manuscript.

## Conflict of Interest

The authors declare that the research was conducted in the absence of any commercial or financial relationships that could be construed as a potential conflict of interest.

## Publisher’s Note

All claims expressed in this article are solely those of the authors and do not necessarily represent those of their affiliated organizations, or those of the publisher, the editors and the reviewers. Any product that may be evaluated in this article, or claim that may be made by its manufacturer, is not guaranteed or endorsed by the publisher.

## Footnotes

1
http://sourceforge.net/projects/trinityrnaseq/files

2
http://wego.genomics.org.cn/cgi-bin/wego/index.pl

3
http://www.genome.jp/kegg/kegg2.html

4
http://www.bioconductor.org/packages/release/bioc/html/topGO.html

## Supplementary Material

Supplementary Table 1 Annotation of unigenes expresses under salinity stress (NT database).

Supplementary Table 2 Annotation of unigenes expresses under salinity stress (NR database).

Supplementary Table 3 **(A,B)** Statistics of predicted unigenes and annotation information statistics for unigenes.

Supplementary Table 4 **(A,B)** The number of spectra, unigenes, peptides, and proteins found in *Panicum* under salinity. The up and downexpression of genes in all salinity treatments.

Supplementary Table 5 Annotation of unigenes expresses according to the cellular component under salinity stress.

Supplementary Table 6 Annotation of unigenes expresses according to biological process under salinity stress.

Supplementary Table 7 Annotation of unigenes expresses according to molecular function under salinity stress.
