# Supplementary material for: Combined Transcriptome and Proteome Analysis to Elucidate Salt Tolerance Strategies of the Halophyte Panicum antidotale Retz
Source: Front Plant Sci. 2021 Nov 2;12:760589. doi: 10.3389/fpls.2021.760589 (PMC8598733; doi:10.3389/fpls.2021.760589)
Supplement: Supplementary Table 1 — Annotation of unigenes expresses under salinity stress (NT database). [file Data_Sheet_1.ZIP › Suppl/Supplimentory Figures.docx]

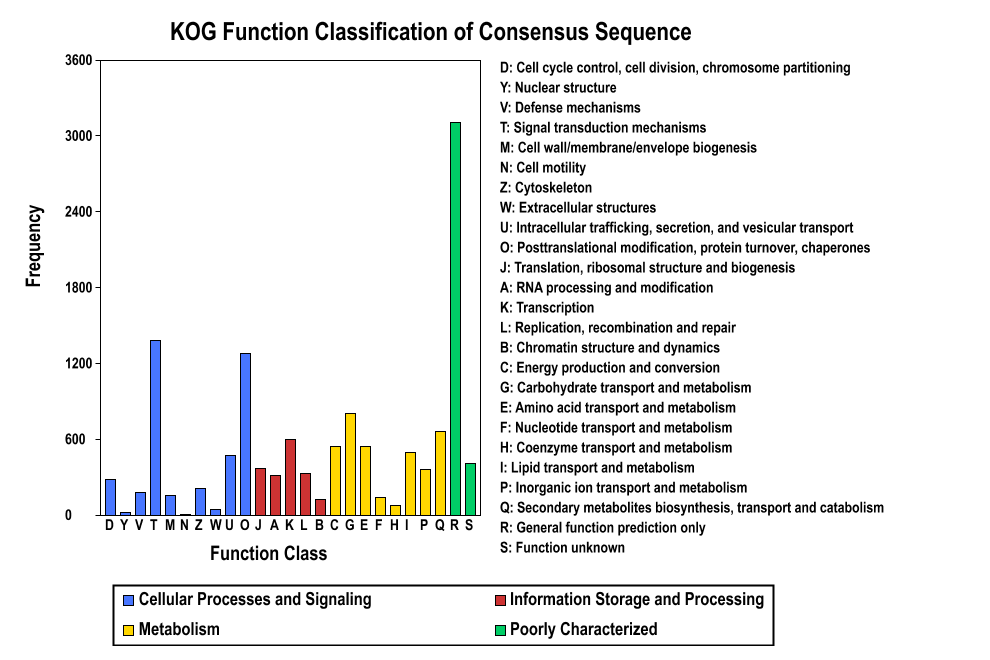


**FigS1** KOG function classification of unigenes observed.


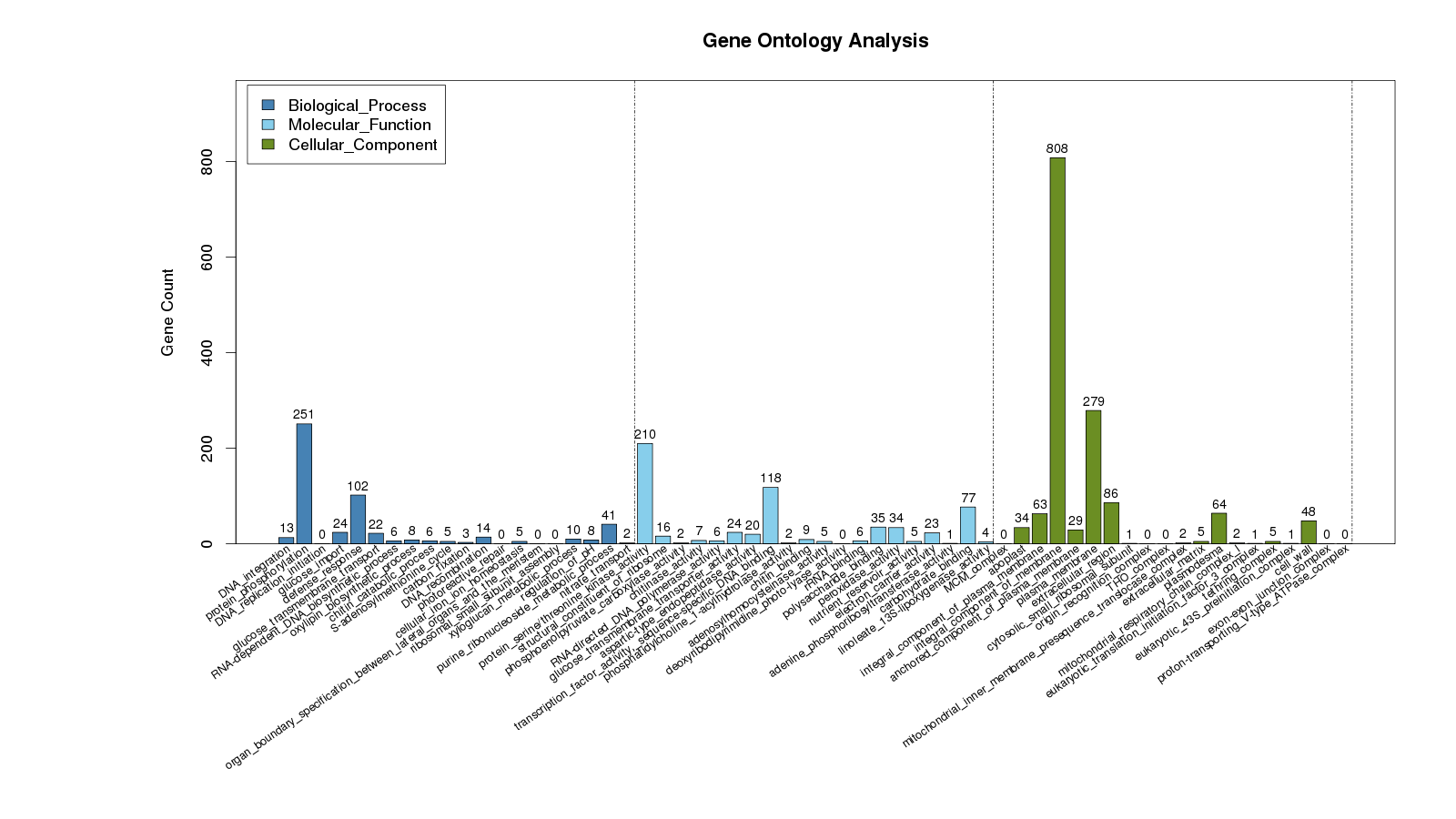


A


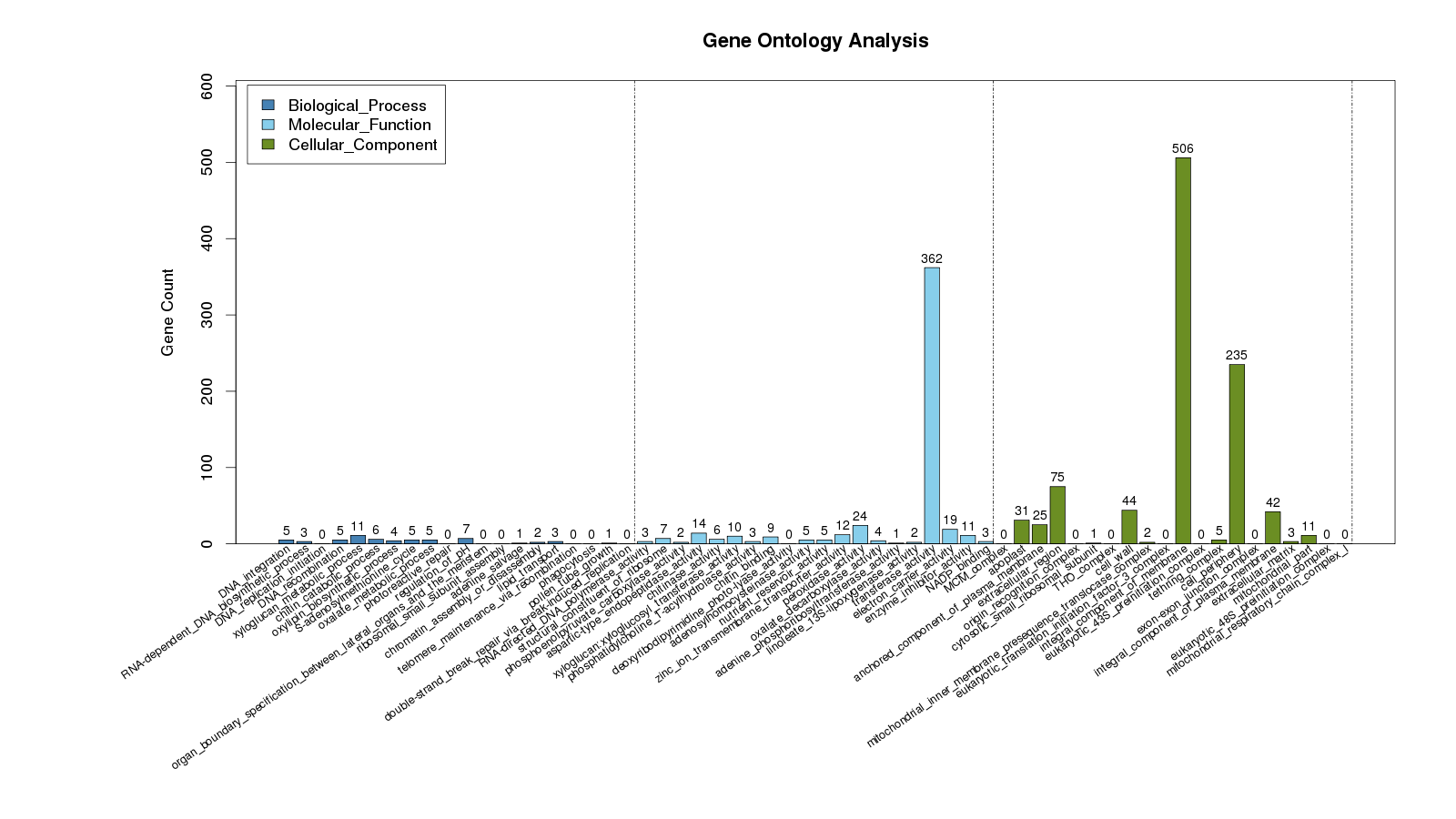


B


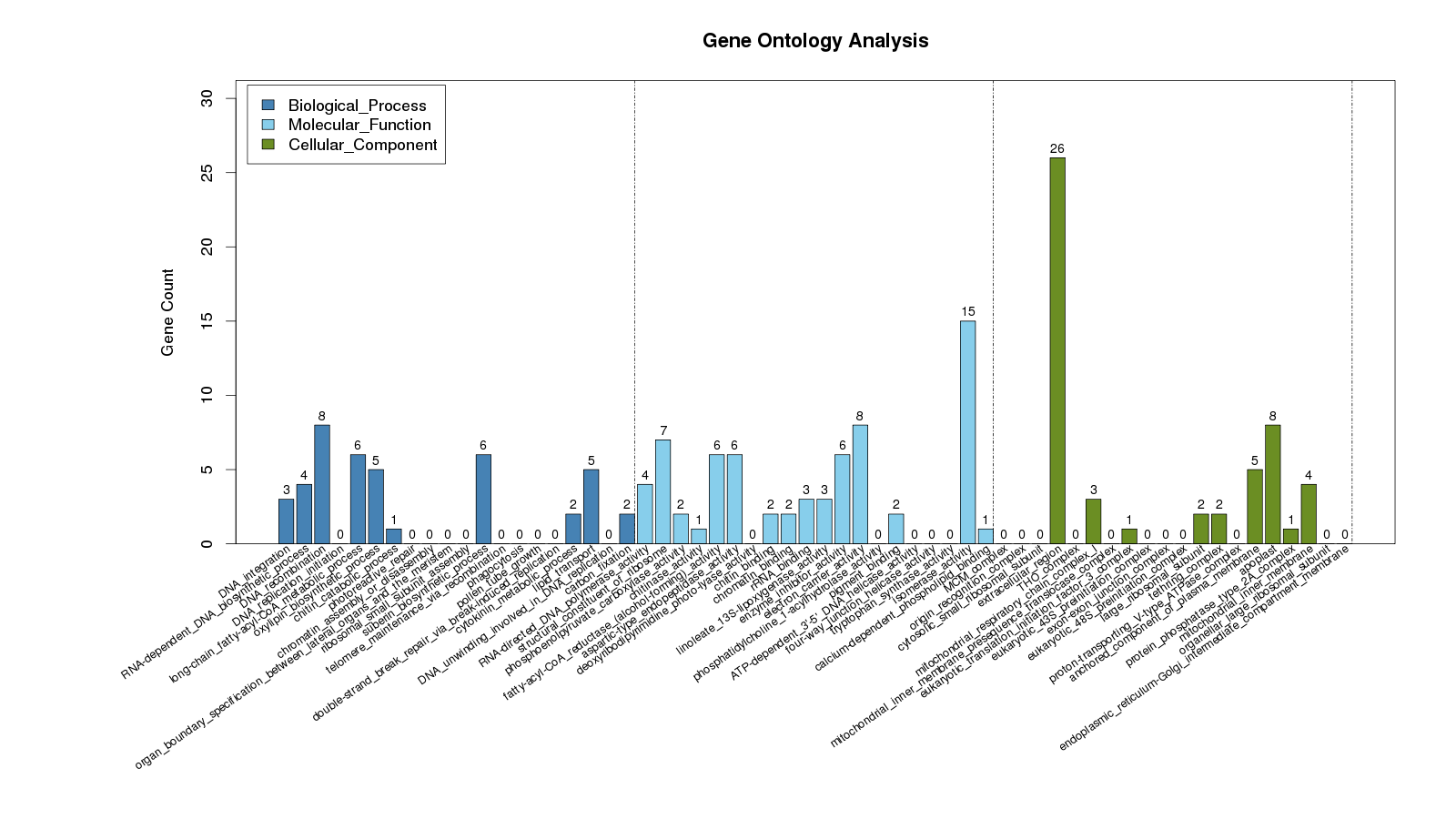


C

**FigS2** GO Classification on the basis of biological process, molecular function and cellular component of differentially expressed genes under salinity. Bar representing number of DEGs recorded in treatments: (A) low salinity / control, (B) high salinity / control, and (C) high salinity / low salinity.


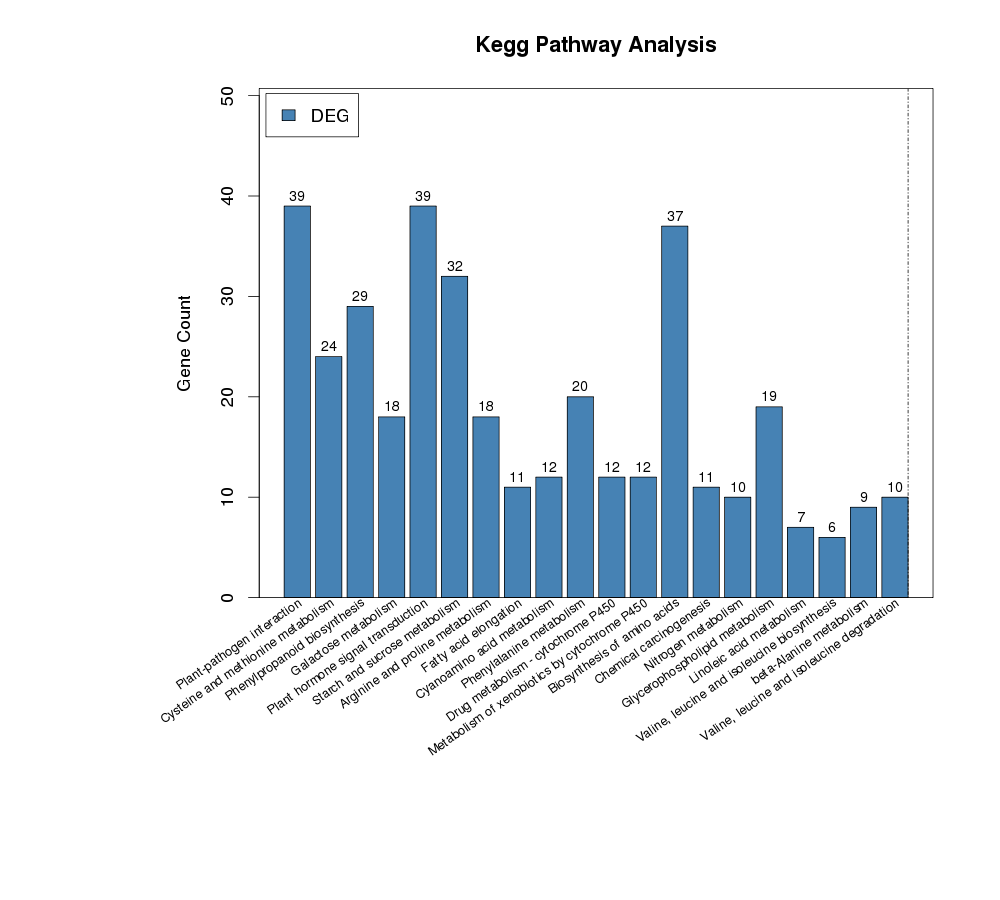


A


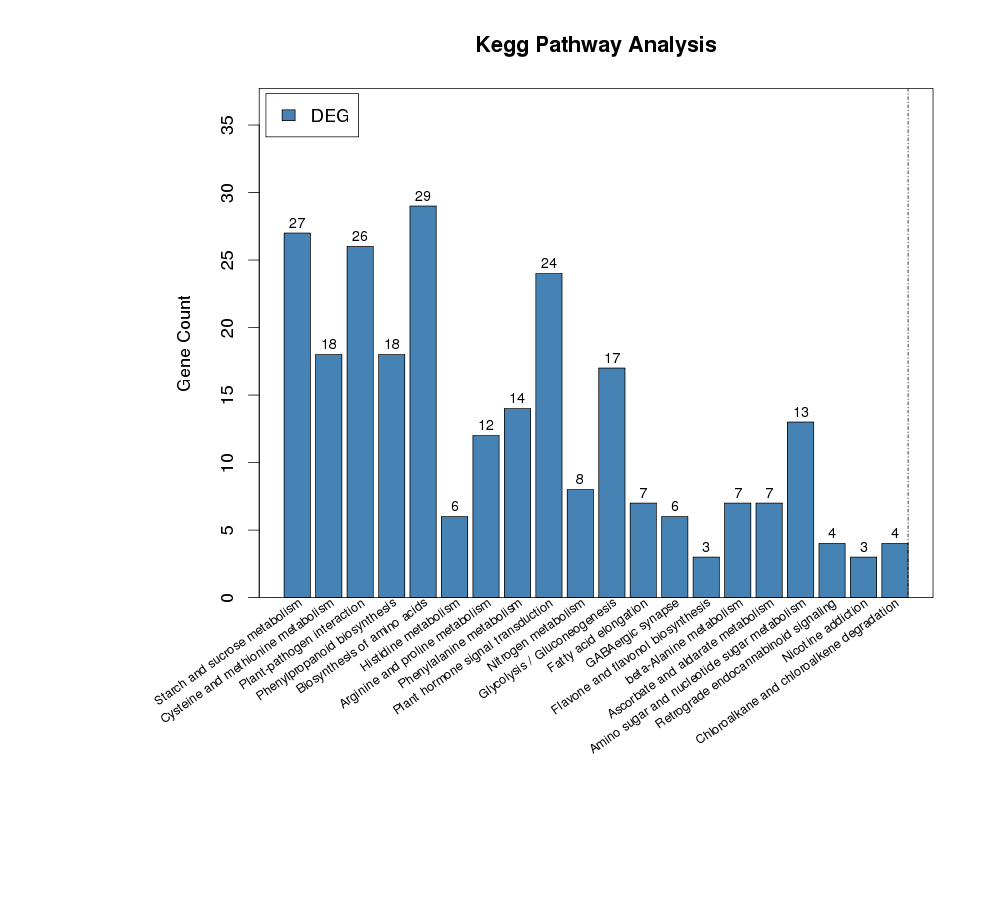


B


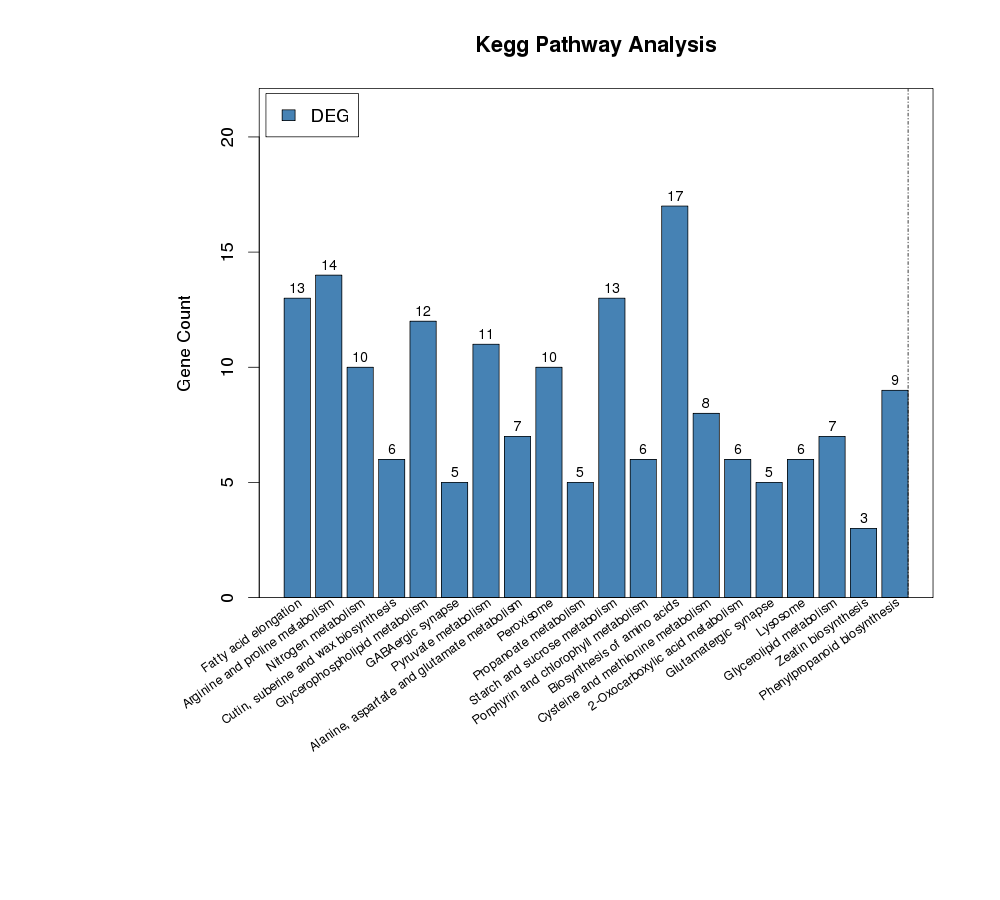


C

**FigS3** KEGG Classification on the basis of biological process, molecular function and cellular component of differentially expressed genes under salinity. Bar representing number of DEGs recorded in treatments: (A) low salinity / control, (B) high salinity / control, and (C) high salinity / low salinity.

**Fig. S4** (i) Scatter diagram of KEGG pathway enrichment for up- and don-regulated DEPs in low salinity compared to control treatment. The top 20 pathways were listed in the plots. The X-axis corresponds to the ratio of DEPs in this pathway to all the genes in this pathway, and the Y-axis represents a diﬀerent pathway. The magnitude of the dots displays gene number, and the q-value is described by the colour classification.

**Fig. S4** (ii) Scatter diagram of KEGG pathway enrichment for up- and don-regulated DEPs in high salinity compared to control treatment. The top 20 pathways were listed in the plots. The X-axis corresponds to the ratio of DEPs in this pathway to all the genes in this pathway, and the Y-axis represents a diﬀerent pathway. The magnitude of the dots displays gene number, and the q-value is described by the colour classification.

**Fig. S4** (iii) Scatter diagram of KEGG pathway enrichment for up- and don-regulated DEPs in high salinity compared to low salinity treatment. The top 20 pathways were listed in the plots. The X-axis corresponds to the ratio of DEPs in this pathway to all the genes in this pathway, and the Y-axis represents a diﬀerent pathway. The magnitude of the dots displays gene number, and the q-value is described by the colour classification.
